# Coupling Low-Frequency Ultrasound to a Crossflow Microfiltration Pilot: Effect of Ultrasonic Pulse Application on Sono-Microfiltration of Jackfruit Juice

**DOI:** 10.3390/membranes14090192

**Published:** 2024-09-11

**Authors:** Herenia Adilene Miramontes-Escobar, Nicolas Hengl, Manuel Dornier, Efigenia Montalvo-González, Martina Alejandra Chacón-López, Nawel Achir, Fabrice Vaillant, Rosa Isela Ortiz-Basurto

**Affiliations:** 1 Laboratorio Integral de Investigación en Alimentos, Tecnológico Nacional de México—Instituto Tecnológico de Tepic, Tepic 63175, Mexico; headmiramonteses@ittepic.edu.mx (H.A.M.-E.); emontalvo@ittepic.edu.mx (E.M.-G.); mchacon@tepic.tecnm.mx (M.A.C.-L.); 2 Institut Agro, Institut de Re-cherche pour le Développement, UMR Qualisud, Université de Montpellier, Université d’Avignon, Université de La Réunion, 34000 Montpellier, France; manuel.dornier@institut-agro.fr (M.D.); nawel.achir@institut-agro.fr (N.A.); 3 Laboratoire Rhéologie Et Procédés, Grenoble INP (Institute of Engineering Université Grenoble Alpes), Centre National de la Recherche Scientifique, Université Grenoble Alpes, 38000 Grenoble, France; nicolas.hengl@univ-grenoble-alpes.fr; 4 Centre de Coopération Internationale en Recherche Agronomique pour le Développement, UMR Qualisud, Agrosavia, Rionegro-Antioquia 054048, Colombia

**Keywords:** coupled system, fouling, pulpy juice, sono-microfiltration

## Abstract

To reduce membrane fouling during the processing of highly pulpy fruit juices into clarified beverages, a crossflow Sono-Microfiltration (SMF) system was employed, strategically equipped with an ultrasonic probe for the direct application of low-frequency ultrasound (LFUS) to the juice just before the entrance to the ceramic membrane. Operating conditions were standardized, and the application of LFUS pulses in both corrective and preventive modes was investigated. The effect of SMF on the physicochemical properties and the total soluble phenol (TSP) content of the clarified juice was also evaluated. The distance of ultrasonic energy irradiation guided the selection of the LFUS probe. Amplitude conditions and ultrasonic pulses were more effective in the preventive mode and did not cause membrane damage, reducing the operation time of jackfruit juice by up to 50% and increasing permeability by up to 81%. The SMF did not alter the physicochemical parameters of the clarified juice, and the measured LFUS energy ranges did not affect the TSP concentration during the process. This study is the first to apply LFUS directly to the feed stream in a pilot-scale crossflow microfiltration system to reduce the fouling of ceramic membranes and maintain bioactive compounds in jackfruit juice.

## 1. Introduction

The health problems associated with consuming ultra-processed foods have promoted the development of new processes to guarantee the safety and preservation of sensory attributes and bioactive compounds in products [1,2]. Microfiltration (MF) is used industrially and has enabled the development of microorganism-free, natural beverages [3,4] without heat treatments that alter their nutritional and organoleptic properties. However, the main challenge in MF is the phenomenon of membrane fouling, caused by the deposition of material from the feed fluid on the membrane surface and/or in the membrane pores. This reduces the permeability and yield of the process at a rate directly proportional to the concentration of solids present in the retained juice [5]). The suspended solids generally responsible for this membrane fouling are residual polysaccharides from enzymatic hydrolysis, originating from the cell wall of fruits as insoluble pectins, cellulose, hemicelluloses, and lignin [6,7]. The concentration of these macromolecules on the membrane surface leads to fouling and, consequently, a decrease in permeate flux during the microfiltration process. To mitigate fouling, the coupling of microfiltration with new technologies that enhance filtration efficiency and performance has been sought, among which is the use of low-frequency ultrasound (LFUS), which stands out [8].

LFUS (corresponding to a frequency range of 20–100 kHz) has multiple applications in fluid food processing, from disrupting tissues or cells to extracting bioactive compounds [9], modifying the texture, viscosity, and structure of compounds of interest [10], and even surface decontamination [11]. These applications are feasible thanks to the cavitation phenomenon, which involves the formation, reduction, and collapse of tiny bubbles generated by an ultrasonic field, resulting in pressure increases that affect food matrices [12]. The ultrasonic energy released by ultrasound-generated cavitation facilitates the cleaning of contaminated surfaces and improves permeate flow by inducing critical physical phenomena in solid–liquid systems, such as microstreaming, acoustic streaming, microjets, microstreamers, and shock waves [13,14].

The impact of LFUS as a pretreatment before MF has been investigated, showing significant improvements in permeate flux performance [15]; it is also effective for membrane cleaning [8]. The indirect application of LFUS, i.e., without direct contact of the probe with the fluid, has been studied [16,17,18,19], but these methods have limitations. The system configuration can affect the cavitation efficiency and may not effectively reduce fouling. Additionally, some methods require removing the membrane from the equipment for cleaning, which can damage the membrane and reduce its service life. The study by Navarrete-Solís [16] showed that applying LFUS external to the filtration housing improved the permeability of the clarified juice without affecting bioactive compounds. However, it was also observed that the targeted application of LFUS pulses external to the filtration housing can cause damage or fractures in mineral filtration membranes. Thus, the probe position is a critical factor in defining the coupling of this technology. Patents US6221255B1 and WO2021105609 mention that LFUS reduces fouling but has limitations due to the location of the LFUS probe, as the probe position does not take advantage of the tangential flow effect to decrease the membrane deposition layer, thereby reducing its efficiency.

To overcome the technological challenges reported in the coupling of these two technologies, a novel and practical design of a Sono-Microfiltration (SMF) pilot is presented in this study. Support that allows the ultrasonic probe to be attached was strategically installed to insert interchangeable ultrasound probes to apply LFUS pulses to the feed fluid just before it entered the filtration membrane. This setup takes advantage of the tangential flow to propagate cavitation phenomena and reduce fouling in the membrane, thereby improving filtration performance. The effect of LFUS applied directly to the fluid to be processed in a filtration pilot to produce clarified beverages has yet to be studied. Therefore, in addition to the innovation of coupling these two technologies, it was of interest to study the effect of applying ultrasonic pulses in corrective and preventive modes at different amplitudes on filtration performance. The physicochemical parameters and TSP of the clarified beverage were also studied to verify the effect of LFUS on jackfruit juice, as the literature is scarce on the use of LFUS in membrane filtration.

This research aimed to couple a low-frequency ultrasound probe to a crossflow microfiltration pilot to evaluate the effect of ultrasonic amplitude and pulses in a new Sono-Microfiltration pilot to obtain a clarified beverage from the pulpy juice of jackfruit (*Artocarpus heterophyllus* Lam).

## 2. Materials and Methods

### 2.1. Raw Material

Ripe jackfruit (Total Soluble Solids TSS 24 ± 2 °Brix) (Romina, Daisy, and Jaiba genotypes) was donated by Frutos Tropicales de la Bahía S.P.R. by R.L. from Ixtapa de la Concepción, Municipality of Compostela, Nayarit, México (21°18′32.2″ N 105°09′22.4″ W). The jackfruits were washed and disinfected with sodium hypochlorite at 200 ppm for 5 min and drained. Bulbs were manually extracted and kept at 4 °C while processing batches with an average batch size of 5 jackfruits (*Artocarpus heterophyllus* Lam.); a total of 80 fruits were processed.

### 2.2. Raw Material Preparation

The previously obtained bulbs were processed in a pulper (Model DP1, MMinoxidable y Servicios, Guadalajara, Mexico) with a 0.8 mm perforated mesh to obtain jackfruit pulp (JP), which was immediately frozen (−20 °C) in approximately 2 kg bags until use. To reduce the viscosity of the pulp obtained (1.950 ± 0.134 Pa·s) and to avoid total fouling of the membrane, for the development of each experiment, it was hydrolyzed in batches of approximately 20 L with pectinase (Pectinase AI, ENZIQUIM, Mexico city, Mexico) and cellulase (Cellulase 10XL, ENZIQUIM, Mexico city, MX) in a 1:1 ratio, added at a concentration of 1% because jackfruit is a highly pulpy fruit. Concerning the amount of pulp, hydrolysis was carried out at 45 °C for 60 min. Due to the high concentration of solids and residual viscosity in the hydrolyzed jackfruit pulp (0.078 ± 0.007 Pa·s), ratios of 1:1 to 1:2 (hydrolyzed pulp/water) were used to demonstrate the effects of LFUS on the flux for the development of the experiments. These samples were referred to as initial feeding jackfruit juice (IFJJ). Finally, the products after SMF are identified as clarified jackfruit juice (CJJ) and retained jackfruit juice (RJJ).

### 2.3. LFUS Coupling to the MF Pilot

**Figure 1:**
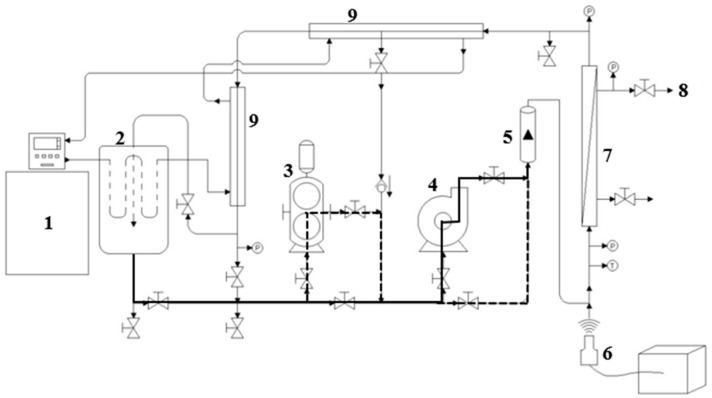
Options for using the pumping system that allow different operating modes to be applied in the Sono-Microfiltration pilot. The solid line indicates the use of the SMF pilot with a centrifugal pump; the dashed line indicates the use of the SMF pilot with a positive displacement pump; the solid and dotted lines indicate the use of both pumps. (1) Recirculating bath; (2) Feed tank (30 L); (3) Positive displacement pump; (4) Centrifugal pump; (5) Flowmeter; (6) Ultrasound probe; (7) Filtration membrane housing; (8) Permeate flow outlet; (9) Tubular heat exchanger.

A tangential sono-filtration (SFT) pilot system was designed to allow the use of microfiltration (Figure 1). The pilot includes a pumping system composed of a positive displacement pump to generate the required pressure and allow for the processing of highly viscous fluids and a centrifugal pump to control the recirculation rate in the pilot. The heat exchange area was increased by using jacketed piping and connected to a recirculation bath with temperature control, preventing overheating of the fluid due to recirculation friction and ultrasonic energy dissipation. A strategically positioned support was coupled at the inlet of the membrane module to insert ultrasonic probes adaptable to different diameters, enabling the application of the LFUS pulses to the feed fluid just before it enters the housing that contains the filtration membrane (Patent registration application, MX/E/2024/041683). This setup leverages the tangential flow to extend the cavitation effects to the membrane surface, keeping the particles in motion and optimizing cavitation phenomena to reduce fouling and increase filtration efficiency. An additional advantage of the SFT design is that the membrane does not need to be removed from the equipment for cleaning, which extends the membrane’s lifetime and increases effective process uptime.

**Figure A1:**
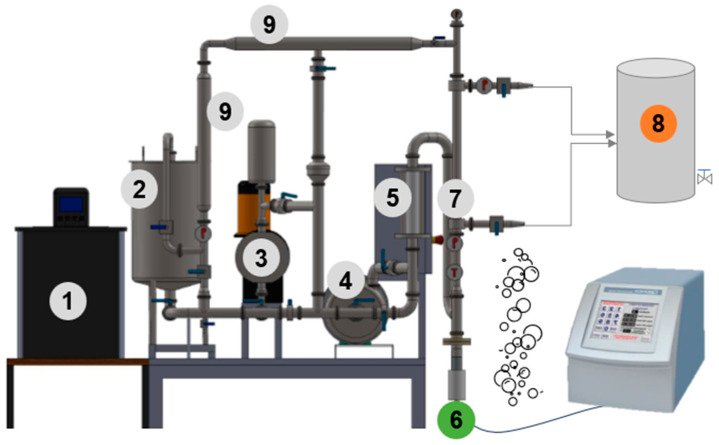
Design of the Sono-Microfiltration pilot. (1) Recirculating bath; (2) Feed tank (30 L); (3) Positive displacement pump; (4) Centrifugal pump; (5) Flowmeter; (6) Ultrasonic probe; (7) Filtration membrane housing; (8) Permeate flow outlet; (9) Tubular heat exchanger.

Each US probe evaluated (1.27 cm and 2.54 cm) was connected to a QSonica generator (Q700 Sonicator, Newtown, CT, USA) with a maximum power (P) of 700 W, a frequency (F) of 20 kHz, and an ultrasonic intensity of 142.4 W·cm^2^ in water as a Newtonian fluid (0.001 Pa·s). Figure 1 shows the main components of the SFT pilot and pilot operation options (Appendix A, Figure A1). For this study, the tubular ceramic microfiltration membranes (TiO_2_) had a length of 58 cm, a diameter of 2.54 cm, 23 multichannels (each with a hydraulic diameter 0.35 cm), a pore diameter of 0.2 µm, and a filtration area of 0.35 m^2^ (Tami industries^®^, Nyons, France). This setup is referred to as the Sono-Microfiltration pilot (SMF).

#### 2.3.1. Membrane Washing and Preparation of the Sono-Microfiltration Equipment

A NaOH solution (10 L) at a concentration of 0.1 N was fed into the system at 80 °C to eliminate all types of organic matter, which was recirculated for 30 min, followed by rinsing with water until neutralized at pH 7. Next, HNO_3_ (10 L, 0.1 N) at 60 °C was recirculated at 60 °C for 15 min to remove any residue and inorganic contaminants, followed by rinsing with water until neutralized at pH 7. Finally, 10 L of water were recirculated with food-grade sanitizer (Tane Citrus^®^, REDBOX, Mexico city, Mexico) at a concentration of 2% for 15 min. The washing was performed with the permeate valve open to remove any residual juice. The water flux was then measured before and after membrane washing.

In all cases, it was confirmed that the water permeability of the membrane was maintained at least at 90%. If necessary, the washing process was repeated.

#### 2.3.2. Membrane Integrity

Membrane integrity was evaluated before starting each experiment, confirming compliance with Darcy’s law [20,21]. Purified water was recirculated at TMP between 0.2 and 2 bar. The permeate flux (L·h^−1^·m^−2^) was calculated, the line equation was plotted, and the value of R^2^ was calculated. The linearity of the results between the increase in transmembrane pressure and permeate flux confirmed adequate membrane integrity. Once the membrane integrity was verified, each experimental run was performed using the jackfruit juice prepared, as mentioned in Section 2.2.

#### 2.3.3. Permeate Flux (Jp)

The permeate flux was determined by monitoring the volumetric flow rate over the filtration area. The results were expressed in L·h^−1^·m^−2^. A pressure of 2.7 bar was applied in all experiments [22].

#### 2.3.4. Volumetric Reduction Ratio (VRR)

The volumetric reduction ratio (*VRR*) was determined by the ratio of the feed volumes (initial volume; *Vi*) and permeate volumes (*Vp* = initial volume − final volume) at a specific time according to the following relationship (1).

(1) $$VRR=\frac{Vi}{Vi-Vp}$$

#### 2.3.5. Membrane Fouling and Loss of Permeability

In cases in which consecutive evaluations were performed to determine the effects of different ultrasonic intensities, the specific fouling was evaluated thanks to the total hydraulic resistance of the membrane R compared to the resistance of the clean membrane Rm (calculated from permeate flux according to Darcy’s law). The initial permeability of each experiment was also considered to plot the percentage of permeability loss during the process. The permeability loss of the membrane after the Sono-Microfiltration process was determined as follows (2).

(2) $$\%\;Loss\;of\;permeability=\frac{Jp\;initial-Jp\;final}{Jp\;initial}\times 100$$

#### 2.3.6. Hermia Model

The Hermia model [23] was determined to identify the membrane fouling mechanisms causing flux decrease during filtration (3). The original ordinary differential equation was solved for four different discrete values of *n*, each value with its blocking mechanism: complete pore blockage (4), standard pore blockage (5), intermediate pore blockage (6), and deposition layer (7) [24].

(3) $$\frac{d^{2}t}{dV^{2}}=k{\left(\frac{dt}{dV}\right)}^{n}$$

Complete pore fouling model (*n* = 2) (4):

(4) $$ln\left(\frac{1}{J}\right)=ln\;\left(\frac{1}{J_{0}}\right)+K_{1}t$$

where *J*_0_ is the initial permeate flux, *J* is the permeate flux at a time *t*, and *K* is a constant whose dimension depends on the values of *n*. In this state, the particle size equals the membrane pore; when the particle reaches the membrane surface, it blocks the pores.

Standard pore-blocking model (*n* = 1.5) (5):

(5) $$\frac{1}{\sqrt{J}}=\frac{1}{\sqrt{J_{0}}}+K_{2}t$$

In this model, the particles are smaller than the diameter of the membrane pores, so they can enter through the pore and adhere to the walls, reducing the pore volume.

Intermediate pore-blocking model (*n* = 1) (6):

(6) $$\frac{1}{J}=\frac{1}{J_{0}}+K_{3}t$$

In this case, the particles are similar to the diameter of the pores, so the particle reaches the membrane and may seal some pores or overlap with other particles. It is similar to completely blocking the pores but is less restrictive.

Deposition layer model (*n* = 0) (7):

(7) $$\frac{1}{J^{2}}=\frac{1}{J_{0}^{2}}+K_{4}t$$

The model represents the case where the particle diameter is larger than the diameter of the membrane pores. The pores are blocked in this case, and the deposition layer is formed on the membrane surface.

### 2.4. Selection of an Ultrasonic Probe Based on the Distance of the Energy Emitted at Different Amplitudes in the SMF Pilot

To visualize the LFUS propagation distance, the temperature field displacement, and the irradiated ultrasonic intensity in the tubing, two probes of different diameters (1.27 cm and 2.54 cm) were measured with a thermal imager (Model TiS40, Fluke Corporation, Everett, WA, USA). The ultrasonic probe to be attached was selected to avoid mechanical damage to the filtration membrane. A 2 × 5 statistical design was applied, evaluating two LFUS probe diameters at five ultrasonic amplitudes (20, 40, 60, 60, 80, and 100%). The treatments were applied for 5 min of operation in the SMF pilot without the membrane installed, with water in the pilot lines and without temperature control, to visualize ultrasonic energy dispersion. For each treatment, the power (W) and ultrasonic energy (J) were recorded, and the ultrasonic intensity (USI) was calculated from the average power generated (W) and the effective area of ultrasonic energy generation of each probe (cm^2^). No calorimetry measurement was performed, so the USI is referred to as the total electricity supplied by the ultrasonic generator.

### 2.5. Application of Ultrasonic Pulses in Corrective Mode with the Selected Ultrasonic Probe to Recover the Permeability of the Filtration Membrane

Ultrasonic pulses were applied in corrective mode to recover the membrane permeability after 45 min of operation in the MF pilot (without LFUS application) to form the deposition layer and to confirm the correct positioning of the probe and the US energy needed to reduce filtration membrane fouling.

Batches of 20 L of jackfruit juice with a pulp/water ratio of 1:1, viscosity of 0.019 ± 0.003 Pa·s, and TSS from 9.6 to 11.4 °Brix were used. Constant MF operating conditions were maintained in all experiments: a transmembrane pressure (TMP) of 2.7 bar, a temperature of 30 °C, crossflow velocity (U) of 6 m·s^−1^, and a volumetric reduction ratio (VRR) of 1 during the first 45 min to induce fouling. A one-factorial design was evaluated by varying amplitudes of 30%, 40%, and 50% at a frequency of 20 kHz using the selected ultrasonic probe to apply the ultrasonic pulses for 1 min (ON) in corrective mode with rest times of 1, 5, and 10 min (OFF). The response variable was the permeate flux (L·h^−1^·m^−2^) both during MF operation (without LFUS application) and the application of LFUS pulses in corrective mode.

### 2.6. Preventive Ultrasonic Pulse Application on Process Time Reduction in the SMF Pilot and Control Parameters

A second experiment was conducted to evaluate the effect of LFUS pulses in the SMF pilot in preventive mode on membrane permeability. For this purpose, the feed flow to the system was started, and LFUS pulses were immediately applied before the transmembrane pressure was applied. The objective was for the LFUS pulses to act preventively, avoiding the formation of the deposition layer and fouling of the filtration membrane. A 2^3-1^ fractional factorial experimental design was applied (Table 1), varying the LFUS parameters of pulse duration (ON time), pulse frequency (OFF time), and US amplitude.

For the experimental runs, hydrolyzed pulp batches were used in a pulp/water ratio of 1:2 with a viscosity of 0.011 ± 0.002 Pa·s. Additionally, MF operation without LFUS application was evaluated as a reference run over 100 min to determine the time required to reach the same VRR under the conditions defined in the design experiments. Clarified and retained juice were obtained in each experiment and kept for physicochemical analysis.

The response variable was the permeate flux during the time required to achieve the VRR of the MF run. Power (W), USI (W·cm^2^), and total volumetric energy applied (J·L) were monitored during the experiments in the SMF pilot with US pulse application. A contour analysis was performed to find the best operating conditions to obtain a higher permeate flux in the SMF pilot with Statistica v12 software.

#### Ultrasonic Energy USE Generated during Sono-Microfiltration

The ultrasonic energy generated by each SMF treatment was monitored on the QSonica Q700 equipment (Qsonica, Newtown, CT, USA) for each 20 L batch to correlate the effect of LFUS with polyphenol stability. The ultrasonic energy (EUS) was calculated by dividing the total energy dissipated (J) per liter of filtered suspension (L).

### 2.7. Evaluation of Physicochemical Properties and Phenolic Compounds of Sono-Microfiltered Jackfruit Juice

The physicochemical parameters and total soluble phenol content were measured in jackfruit pulp, clarified jackfruit juice, and retained jackfruit juice (products of each run of the experimental design mentioned in Section 2.6).

#### 2.7.1. pH, TA, and TSS Determination

The pH was determined according to the AOAC 981.12 method. The AOAC 942.15 method was used to measure the titratable acidity (TA), by potentiometric titration with NaOH 0.1 mol∙L^−1^ up to a pH of 8.1, of a 5 g homogenized sample mixed with 25 mL of deionized water. The results were reported in g of citric acid∙kg^−1^ of the sample. Total soluble solids (TSS) were determined by the AOAC (1990) method using an adequately calibrated digital refractometer MASTER-53M (ATAGO CORPORATION, Minato-ku, Tokio, Japan) after centrifugation at 5000× *g* for 10 min to remove suspended solids.

#### 2.7.2. Total Reducing Sugars Determination (TRS)

Freeze-dried jackfruit bulbs were homogenized using a commercial blender (NUTRIBULLET^®^, Los Angeles, CA, USA). TRS was measured according to the NMX-V-006-NORMEX-2005. The results were expressed in g∙kg^−1^.

#### 2.7.3. Moisture and Dry Matter Determination

Moisture was determined on a Thermobalance (Model MB23, OHAUS CORPORATION, Guiping Road Shanghai, China). A total of 2 g of jackfruit pulp was placed in an aluminum tray at 105 °C for 45 min. Then, the moisture percentage was obtained. The remaining percentage was taken as a measurement of the dry matter in the sample.

#### 2.7.4. Viscosity Determination

Viscosity was determined with a TA Instrument HIBRID HR1 (TA Instrument, New Castle, DE, USA) temperature-controlled rheometer at 25 °C, with a flow peak hold for 300 s and a shear rate of 50 s^−1^. For JP, the geometry of a plate and cone 60 mm in diameter and with an angle of 2° was used with a sample volume of 23 mL at a gap of 1 mm. For the CJJ and RJJ, a Couette geometry diameter of 28.04 mm, length of 41.88 mm, and sample volume of 22.5 mL at a gap of 6 mm was used. The data were obtained using TRIOS v3 Software (TA Instrument).

#### 2.7.5. Turbidity Determination

Turbidity was determined with a Turbidimeter HACH (Model 2100Qis, HACH COMPANY, Guiping Road Shanghai, China), calibrated with standard solutions (10 to 800 NTU). Results were reported as Nephelometric Turbidity Units (NTU).

#### 2.7.6. Total Soluble Phenols (TSP)

TSP was determined with the Folin–Ciocalteau method [25]. The extraction of TSP was carried out with 5 g of jackfruit pulp and 30 mL of 90% methanol for 2 h under stirring at 4000 rpm. The sample was centrifuged for 8 min at 6000× *g* at 4 °C (Sigma 2-16KL, Osterade am Harz, Germany). The supernatants of the mixture were recovered by filtration (Whatman, Maidstone, UK) and evaporated in a vacuum at 30 °C. These phenolic extracts were stored at −20 °C until analysis. Results were expressed as grams of gallic acid equivalent (GAE) per kg.

### 2.8. Statistical Analysis

The results were analyzed using an ANOVA with a significance value of *p* < 0.05 with Statistica v12 software. All analyses were performed in triplicate.

After this statistical analysis, it was possible to perform the correct selection of the HIUS probe and ultrasound conditions and, subsequently, to confirm the expected effect when assembled in the new SMF pilot for measuring control parameters in the pilot.

## 3. Results and Discussion

### 3.1. Selection of an Ultrasonic Probe Based on the Dissipation of the Energy Emitted at Different Amplitudes in the SMF Pilot

**Figure 2:**
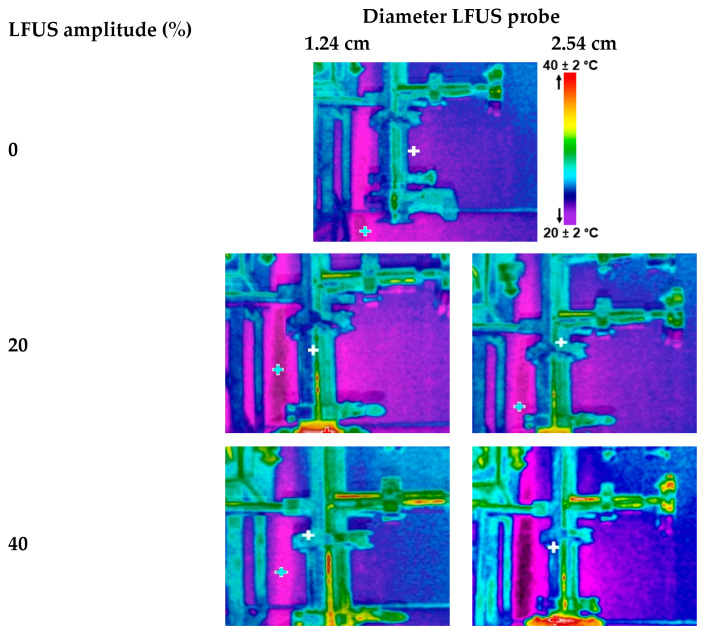

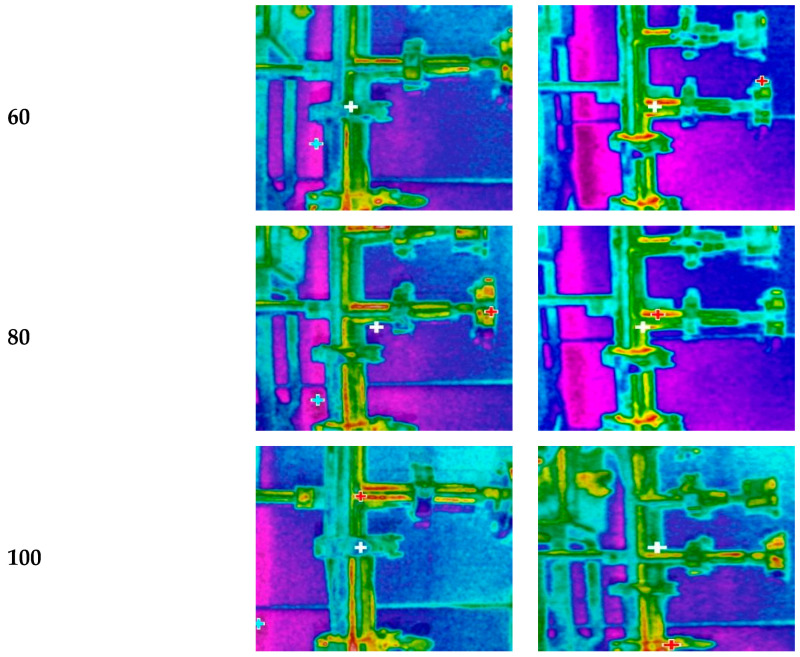
Thermal imaging of ultrasonic energy irradiation from two probes at different amplitudes of low-frequency ultrasound (LFUS) (without membrane filtration, U = 6 m·s^−1^).

The ultrasonic probe was placed in a 3.81 cm diameter pipe, positioned at 35 cm from its tip to the inlet of the filtration housing to evaluate the effectiveness of ultrasonic energy irradiation in preventing particle deposition on the membrane surface (Figure 1, points 6 to 7). A thermographic camera was used to identify the areas where temperature changes occurred (Figure 2), which are directly related to the US energy distribution. Indeed, US irradiation can cause temperature increases due to energy dissipation and the cavitation effect. The zones in brighter colors (red, yellow, and orange) indicate warmer temperatures (higher infrared radiation and higher energy). In contrast, the blue and green zones represent cooler temperatures (lower infrared radiation and lower energy).

A statistically significant upward increase (*p* < 0.05) in the energy irradiation distance was observed with LFUS pulses at amplitudes between 20% and 40%, with a maximum distance of 20 cm. There were no statistically significant differences (*p* > 0. 05) in irradiation distance between amplitudes of 60 to 100%. Statistically significant differences (*p* < 0.05) were also observed in the energy irradiation distance between the two ultrasound probes at low amplitudes of 20% and 40%, but no significant differences (*p* > 0.05) were found above 60% amplitude. For both ultrasound probes, the power and USI increased with the applied ultrasound amplitude, promoting cavitation and heat transfer [26]. However, in the areas where there is no ultrasonic energy dissipation, the force generated by the recirculation of the fluid recirculation in the Sono-Microfiltration system, with a flow velocity of 6 m·s^−1^, keeps the particles in constant motion, thereby reducing the probability of deposition on the membrane surface.

Statistical analysis shows significant differences (*p* < 0.05) between the USI of the two probes evaluated (Table 2), which was corroborated by the thermographic images, as they showed a lower intensity of warm colors in the 2.54 cm probe. Both studies indicate that the energy irradiation distance in the medium is not influenced by the probe used, suggesting uniform energy distribution in the medium. However, the USI generated by each ultrasonic probe did show statistically significant differences (*p* < 0.05). Specifically, it was observed that the smaller the probe diameter, the higher the ultrasonic energy generated, a relationship that had been observed previously [27].

Ultrasonic energy effectively removes particles on the filtration membrane [28], increasing permeability in filtration systems. Therefore, combining ultrasonic energy irradiation with fluid recirculation in an SMF system can be an effective tool in preventing particle deposition on the membrane surface and maintaining filtration performance. However, LFUS has also been reported to cause damage to the filtration membrane, even indirectly [14,24]. To avoid damage to the ceramic membrane and the bioactive compounds in jackfruit juice due to the effects of the generated ultrasonic intensity, the 2.54 cm probe was selected for the subsequent steps.

### 3.2. Application of Ultrasonic Pulses in Corrective Mode to Recover the Permeability of the Filtration Membrane

**Figure 3:**
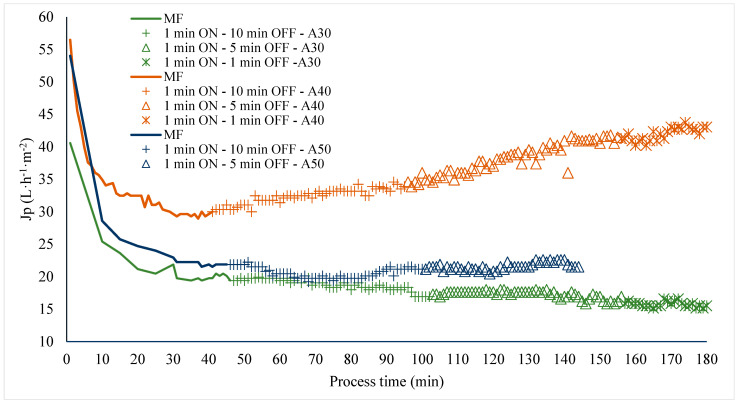
Effect of LFUS application in corrective mode during the processing of jackfruit juice. Pulp/water ratio of 1:1 at VRR = 1 (ultrasonic diameter probe 2.54 cm, membrane Tami, dpore = 0.2 μm, TMP = 2.7 bar, T_feed_ = 30 °C, and U = 6 m·s^−1^). ON: application of LFUS, OFF: without LFUS, A: LFUS amplitude, MF: Microfiltration.

After 45 min of operation at a VRR of 1 (with permeate returned to the feed tank) and without application of LFUS, decreases in the initial flux of 50.4, 73.1%, and 49.5% were recorded (Pulp/water ratio of 1:1, ultrasonic probe diameter of 2.54 cm, membrane Tami, dpore = 0.2 μm, TMP = 2.7 bar, T_feed_ = 30 °C, and U = 6 m·s^−1^), indicating membrane fouling. The variation in initial flux is attributed to differences in the composition batches processed for this study. The application of LFUS pulses in corrective mode at amplitudes of 30%, 40%, and 50% showed (Figure 3) that a 40% amplitude (USI of 26.5–28.5 W·cm^2^ and power of 130–140 W) resulted in the highest recovery of permeate flux. As the LFUS application times were shortened (OFF time), a gradual increase in the corrective ultrasound effect on permeate flux recovery was observed. For the application of corrective pulses at 40% amplitude, a final recovery of 70% was achieved after applying LFUS with 1 min of ON pulses and 1 min of OFF pulses. Conversely, when applying corrective pulses at 30% amplitude, a decrease in flux was observed with a longer pulse application time, indicating lower membrane permeability and no positive effect of LFUS. This suggests that at this amplitude, the USI of 12.6–16.3 W·cm^2^ and power of 62–80 W were insufficient to induce effective cavitation, reduce fouling, or clean the solid deposition layer on the membrane, even with pulses of 1 min ON and 1 min OFF.

**Figure 4:**
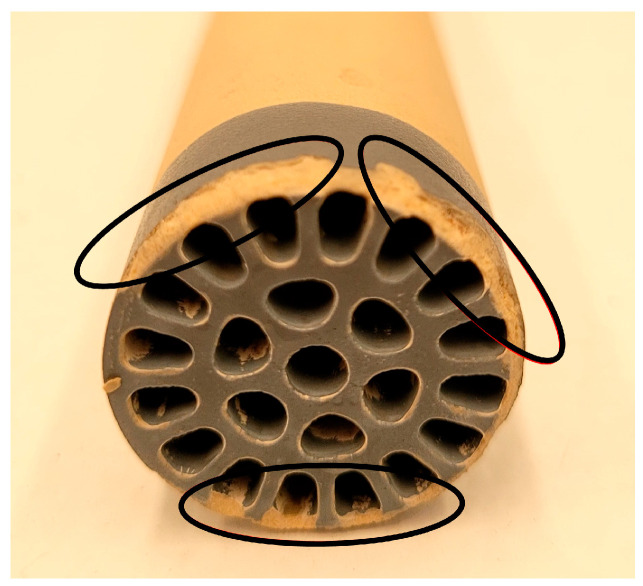
Multichannel ceramic membrane (0.2 μm) with damage caused by applying a 50% LFUS amplitude, an ultrasonic intensity of 21.2–31.2 W·cm^2^, and 104–153 W power.

In the evaluation at 50% amplitude, no significant effect (*p* > 0.05) was observed on the permeate flux with the ultrasonic pulses; instead, a constant permeate flux was observed. Furthermore, the test could not be completed at 50% amplitude because ultrasonic cavitation in the pilot generated turbulence and increased pressure, which ejected the ultrasonic probe. This ruled out the use of this amplitude with the evaluated probe, as it was found that the applied USI of 21.2–31.2 W·cm^2^ and power of 104–153 W caused damage at the inlet end of the filtration housing, where the ultrasonic pulses impacted the membrane (Figure 4). The results demonstrated that although an increase in flux was observed due to the effect of LFUS at 40% amplitude, the 1:1 dilution ratio represents a concentrated beverage with a viscosity of 0.019 ± 0.003 Pa·s and a TSS concentration of 9.6 to 11.4 °Brix, which limited the pilot’s ability to operate for VRRs higher than 1.3 (180 min) due to membrane fouling and a threefold increase in the initial viscosity of the retained juice. This effect can be explained by the accumulation of solids on the membrane surface and residual pectin, which can form agglomerates that increase viscosity due to applied LFUS, as previously reported for ultrasonic processing in other fruits such as mango [29] and strawberry [30].

Based on these results, it was determined that applying LFUS at an amplitude higher than 50% (with USI of 21.2–31.2 W·cm^2^ and power of 104–153 W) causes damage to the membrane. LFUS is more effective when applied in shorter pulses with less time between pulses (OFF time) due to the concentration of solids on the membrane surface, which helps to increase permeability. However, this raises the question of whether applying LFUS in preventive mode from the beginning of the SMF process could achieve better filtration performances. After these results with the 1:1 (*w*/*v*) hydrolyzed pulp/water ratio, an adverse effect of LFUS pulses in corrective mode on permeate flux recovery was observed. Consequently, amplitudes higher than 40% were ruled out to avoid damage to the ceramic membrane and to preserve its useful life. The viscosity of the juice was reduced with a pulp/water ratio of 1:2 to identify the effects of applying LFUS pulses at a 30% amplitude.

### 3.3. Effect of Preventive Ultrasonic Pulses on the Reduction of Process Time in the SMF Pilot

Crossflow microfiltration of hydrolyzed jackfruit juice diluted in water at a 1:2 (*w*/*v*) ratio was performed without ultrasound for 100 min (reference run); during this time, a VRR of 1.85 was achieved. This value was used as a reference to evaluate the reduction in process time by applying ultrasonic pulses in preventive mode, which were applied from the beginning of the SMF pilot operation.

**Figure 5:**
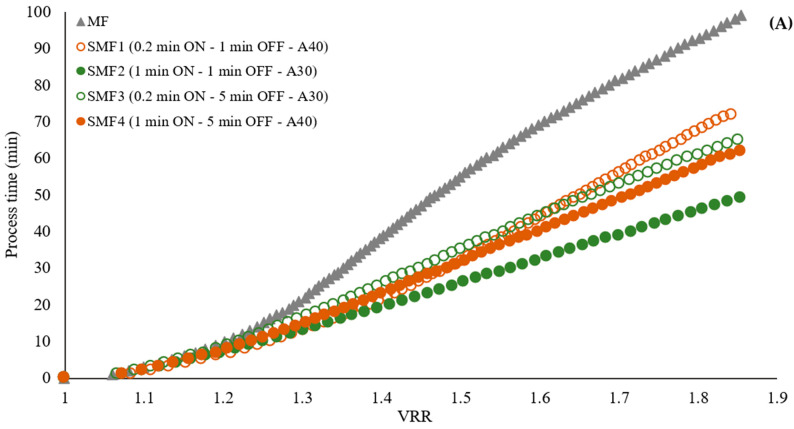

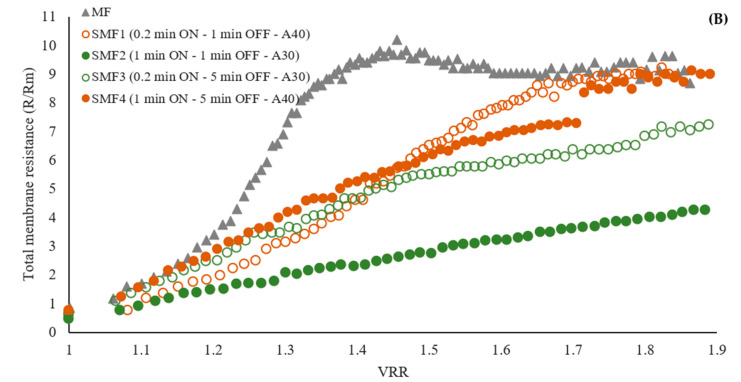
Effect of LFUS application on (**A**) the volumetric reduction ratio (VRR) during the SMF process of jackfruit juicing on process time and (**B**) total membrane resistance to the VRR (ultrasonic diameter probe 2.54 cm, membrane Tami, dpore = 0.2 μm, TMP = 2.7 bar, T_feed_ = 30 °C, and U = 6 m·s^−1^). ON: LFUS application; OFF: without LFUS application; A: LFUS amplitude.

**Figure A2:**
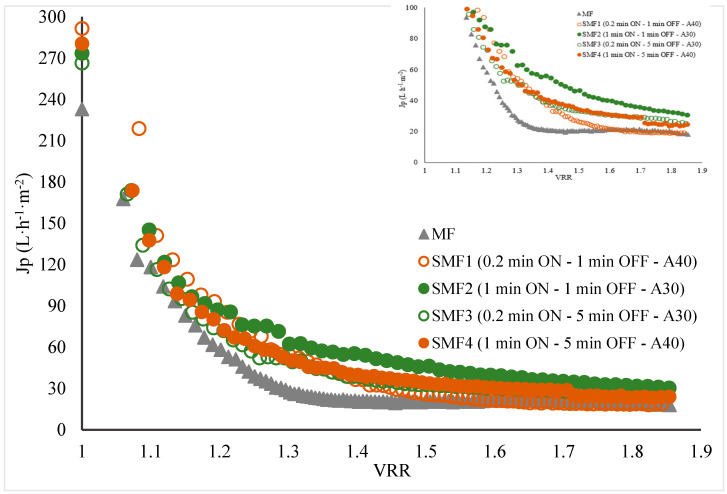
Volumetric reduction ratio (VRR) vs. permeate flux (Jp) during the clarification of jackfruit juice by Sono-Microfiltration.

A reduction in process time of 50%, 47%, 35%, and 28% was observed to achieve the reference VRR when applying the SMF2, SMF4, SMF3, and SMF1 treatments, respectively (Figure 5A and Appendix B Figure A2). The more effective treatments were SMF2 (1 min ON—1 min OFF—30% amplitude) and SMF4 (1 min ON—5 min OFF—40% amplitude). These results demonstrate the effectiveness of the SMF pilot’s performance, showing that by coupling the ultrasonic probe directly to the fluid and applying preventive pulses, processing time was effectively reduced by up to 50%.

**Figure 6:**
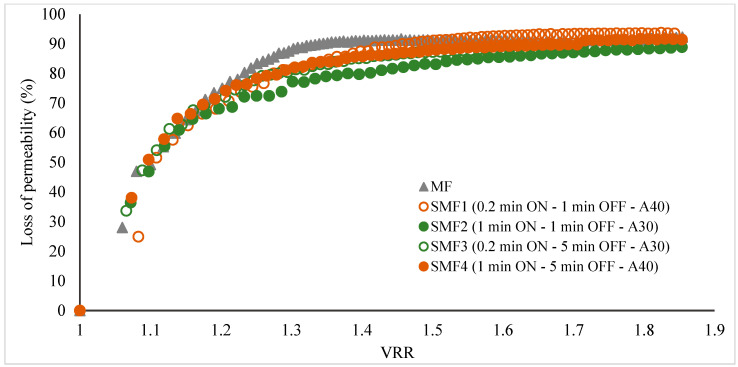
Effect of low-frequency ultrasound application on the decrease in membrane permeability during Sono-Microfiltration processes of clarified jackfruit juice at VRR = 1.85 (ultrasonic diameter probe 2.54 cm, membrane Tami, dpore = 0.2 μm, TMP = 2.7 bar, T_feed_ = 30 °C, and U = 6 m·s^−1^). ON: LFUS application; OFF: no LFUS application; A: LFUS amplitude.

Flux reduction was observed to follow a similar trend in the first 10 min of the process, with a 71–75% decrease in flow. As the VRR increased during the filtration of pulpy jackfruit juice, there was a corresponding increase in the total membrane resistance (Figure 5B). This increase was more pronounced in the microfiltration (MF) treatment, indicating a faster accumulation of particles on the membrane compared to the treatments with LFUS. This resulted in increased resistance due to fouling, adversely affecting the filtration system’s performance. The SMF2 treatment exhibited the lowest R/Rm ratio, indicating that the LFUS application conditions of 1 min ON—1 min OFF at 30% amplitude effectively reduced membrane fouling. According to the results obtained by applying our data to the Hermia model, this effect is primarily caused by the formation of a deposition layer and pore blocking in both MF and SMF treatments (Table 3), as the values are closest to 1. This outcome is attributed to the high turbidity and solid concentration in the juice, which leads to rapid membrane fouling and a decrease in permeate flux (Figure 6). This effect is a commonly identified mechanism explaining flux reduction in membrane filtration [31,32], where constant fouling was observed in MF from a VRR of 1.3. Meanwhile, in the SMF treatments, a gradual reduction may be generated due to increases in viscosity and solid accumulation rather than fouling, as seen in MF. This study is the first to demonstrate the potential of coupling LFUS with this type of application. Further studies at higher VRRs and during longer operating periods will need to be conducted to better evaluate the processes at an industrial level in future reports.

In terms of process performance, MF without LFUS pulses showed a permeate flux of 21.0 ± 0.9 L·h^−1^·m^−2^ after 30 min of processing, which was the minimum value observed under the operating conditions evaluated in jackfruit juice diluted in a pulp/water ratio of 1:2, with a viscosity of 0.011 ± 0.002 Pa·s and turbidity of 11,533 ± 143 NTU. In comparison, the SMF2 treatment presented the highest flux of 39 L·h^−1^·m^−2^, demonstrating an 81% increase in performance and a 50% reduction in processing time due to the use of LFUS. Urošević et al. [33] reviewed the development of microfiltration in fruit juices such as banana, pineapple, blackberry, watermelon, sugar cane, prickly pear, passion fruit, and raspberry, concluding that effective processes were obtained with flux starting at 30 L·h^−1^·m^−2^ at a TMP of 1 to 3 bar. Vaillant et al. [22] reported that in addition to factors such as membrane pore size, the TMP applied, and the operating conditions, the process performance in MF is affected when working with pulpy juices, such as banana and blackberry, which have high turbidity, similar to the jackfruit used in this study. The jackfruit presented high turbidity and viscosity (0.011 ± 0.002 Pa·s and 11,533 ± 143 NTU in hydrolyzed jackfruit pulp and diluted in a pulp/water ratio of 1:2). Hammad et al. [34] reported permeate flux between 30 to 40 L·h^−1^·m^−2^ under conditions similar to those of this investigation (membrane pore size 0.2 µm, T: 30 °C, TMP 2.6 bar, and U: 6 m·s^−1^) for citrus juice with an initial viscosity of 0.0025 Pa·s, which is 4.4 times less than the viscosity of initial jackfruit pulp (0.011 ± 0.002 Pa·s). In other pulpy juices, such as mango, passion fruit, tangerine, naranjilla, and blackberries, permeate flux values of 40 to 70 L·h^−1^·m^−2^ have been reported (recirculation speed: 7 m·s^−1^, pore diameter of 0.2 µm, and TMP of 1.5 bar), with volumetric reduction ratios ranging from 1.3 for mango juice to 3.5 for tangerine and pineapple juice [35].

Jackfruit, being a highly pulpy fruit with a viscosity (1.950 ± 0.134 Pa·s) similar or greater than that reported in other fruits such as mango (2.064 ± 0.197 Pa·s) [36], pineapple (0.021 ± 0.001 Pa·s) [37], and pomegranate fruit (1.660 ± 0.163 Pa·s) [38], generally exhibits lower operational performance in a microfiltration process. However, these results show that directly coupling LFUS to the pilot system positively affects the feed flow before entering the filtration membrane, thereby improving process performance. The hypothesis is that the applied ultrasonic energy disperses particles larger than the membrane’s pore size, reducing the formation of the deposition layer and allowing permeability to be maintained with constant flux, thus shortening the process operation times. The LFUS probe diameter and the effective distance between the probe and the membrane surface mainly define the impact of LFUS on deposited particles. Of the four phenomena that explain the impact of ultrasound on membrane cleaning in a filtration system [39,40], microstreamers and acoustic streaming are the most likely to be generated during the SMF process. These two phenomena radiate energy over a distance of several centimeters from the tip of the probe, while microjets and microstreaming act only some micrometers from the tip of the ultrasonic probe, and thus cannot radiate sufficient energy to the membrane in the SMF system configuration tested. Consequently, the SMF process can be more effective when fruit juices have lower viscosity and turbidity than jackfruit juice. This study is the first to validate the effectiveness of the SMF pilot scale with a highly pulpy juice, demonstrating the effects of LFUS. However, this pilot equipment needs to be tested with other juices to fully demonstrate the benefits of this innovation. In this specific case of pulpy jackfruit juice, it has been shown that ultrasound improves the microfiltration process by enhancing permeate flux. Therefore, it was possible to demonstrate that LFUS, when coupled with MF, represents a promising technology for processing highly pulpy fruits such as jackfruit.

**Figure 7:**
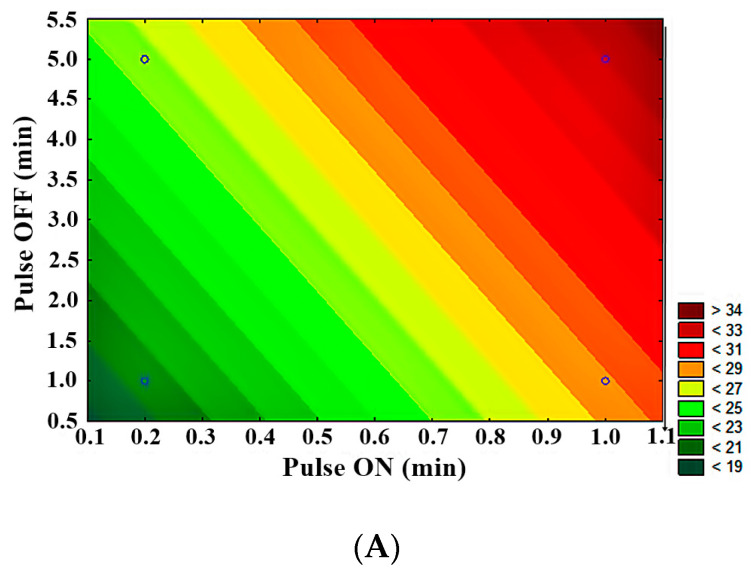

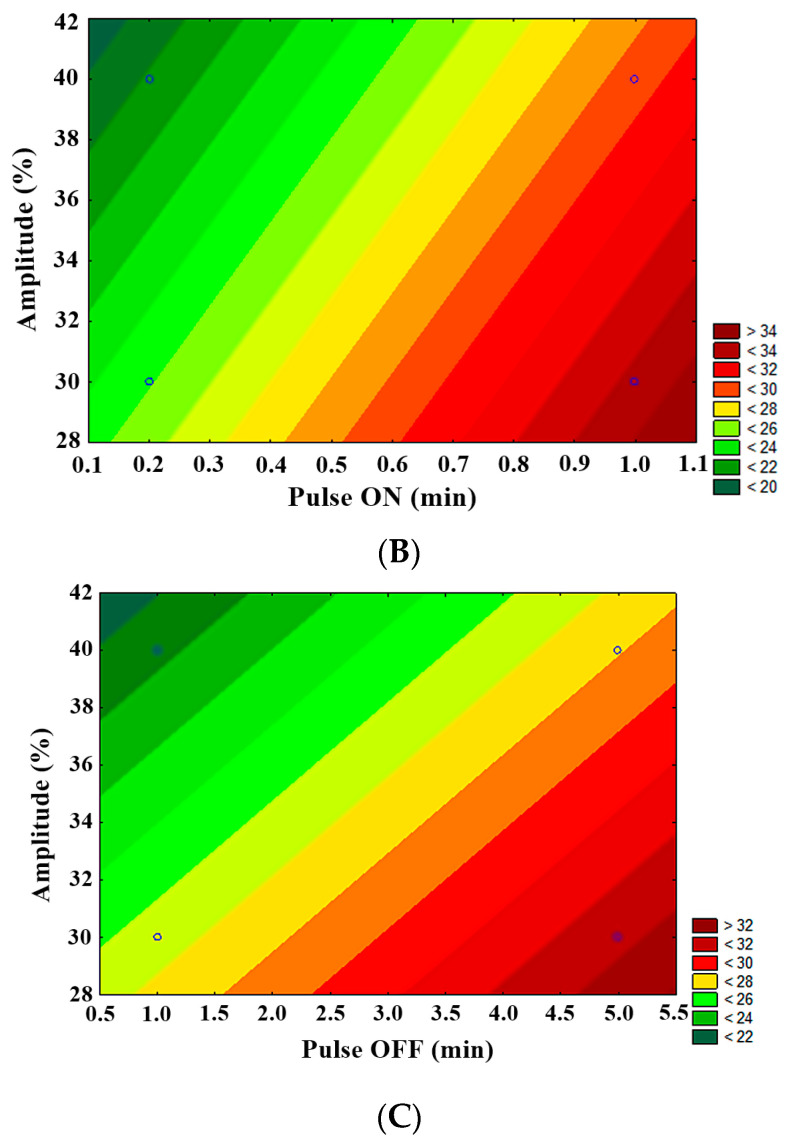
Contour plots of 2^3−1^ fractional factorial design in SMF pilot in jackfruit juice 1:2. (**A**) Pulse OFF vs. Pulse ON, (**B**) amplitude vs. pulse ON, (**C**) amplitude vs. pulse OFF.

Statistical analysis showed significant differences (*p* < 0.05) in the evaluation of LFUS parameters in achieving higher permeate flux. LFUS pulse application (pulse ON) was most conducive to the highest permeate flux under the applied operating conditions, followed by ultrasonic pulse application frequency (pulse OFF) and the percentage of ultrasound amplitude. The contour plots (Figure 7) show that the following conditions resulted in the highest flux:

1. Lower amplitude—higher pulse ON (30% amplitude—1 min ON)
2. Lower amplitude—higher OFF pulse (30% amplitude—5 min OFF)
3. Higher pulse ON—higher pulse OFF (1 min ON—5 min OFF)

The statistical analysis of the fractional factorial design identified the optimal operating conditions for the Sono-Microfiltration pilot as 1 min ON—5 min OFF at 30% amplitude at a VRR of 1.85. This was one of the four treatments that were not directly evaluated due to the fractional factorial analysis nature of the analysis. In validating these optimal Sono-Microfiltration process conditions (data included in a future publication), no statistically significant differences were found compared to the results of the SMF2 treatment. However, there is a reduction in operating costs, which is highly desirable in pilot and industrial-level processes.

This trend on the effect of US pulses has been reported in previous studies [41,42,43]. Here, short pulses (<5 s) were found to improve membrane cleaning in wastewater treatment. Maskooki et al. [44] observed that increasing permeate flux at short LFUS application intervals enhances the cavitation effect, creating greater turbulence around the membrane system. However, existing research on the impact of sonication on flux recovery of microfiltration membranes typically reports an increase in flux with ultrasound powers around 300 W [45,46,47,48]. In this study, the applied power ranged between 70 and 155 W. Although the literature generally supports the effectiveness at shorter pulse application times, it is important to note that the comparisons are often made with processes such as ‘Backflushing’ or backwashing of membranes combined with ultrasound. Therefore, the fouling conditions of the membrane can vary depending on the specific microfiltration process used. In most cases, the application of ultrasound in a filtration system has been studied primarily for cleaning membranes, particularly in wastewater treatment [44,49].

### 3.4. Effect of SMF on the Physicochemical Properties and Phenolic Compounds of Microfiltered Jackfruit Juice

The physicochemical characterization of fresh jackfruit pulp (Table 4) showed values within the ranges reported for this fruit at a ripeness suitable for consumption [50,51]. Galvez & Dizon [52] reported 77.30% moisture in the EVIARC Sweet variety and 75.96% in the AES-2 variety, both from the Philippines. Goswami and Chacrabi [51] evaluated five different cultivars from various regions of Bangladesh, characterized by color (whitish yellow, deep yellow, or light yellow) and texture (firm, very soft, or intermediate), and found moisture values ranging from 79.62 to 84.44%. Navarrete-Solis et al. [53] reported a moisture content of 74.92% in jackfruit from the same area. In fruits originating from Asia, pH values ranging from 4.38 to 5.72 and titratable acidity between 0.12 and 1.04%, expressed as citric acid, have been reported [54,55]. The concentration of TRS in this study was similar to these reports, with values between 29.4 and 81.9 g·kg^−1^ [52,53,54,55]; however, other studies have found values as high as 95.0 g·kg^−1^ [56].

The physicochemical characterization of the SMF products, including the permeated jackfruit juice (PJJ) and the retentate (RJJ), is presented in Table 4. Statistically significant differences in moisture content (*p* < 0.05) were observed, as expected, due to the concentration process that separates juice components. However, pH, titratable acidity, and total reducing sugars did not show statistically significant differences (*p* < 0.05). These results are consistent with those reported by García and Rodríguez [57], who studied the effect of crossflow microfiltration on the physicochemical characteristics of clarified mango juice, demonstrating that the microfiltration process did not alter the physicochemical properties.

**Figure 8:**
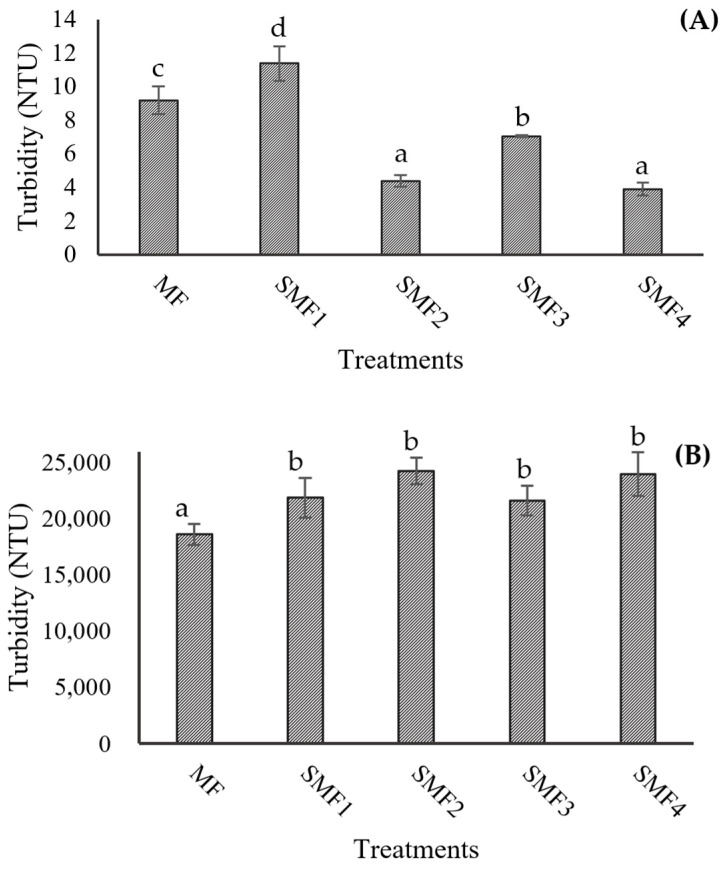
Effect of Sono-Microfiltration (SMF) on the turbidity of (**A**) clarified jackfruit juice (CJJ) and (**B**) retained jackfruit juice (RJJ). Different letters show statistically significant differences.

**Figure 9:**
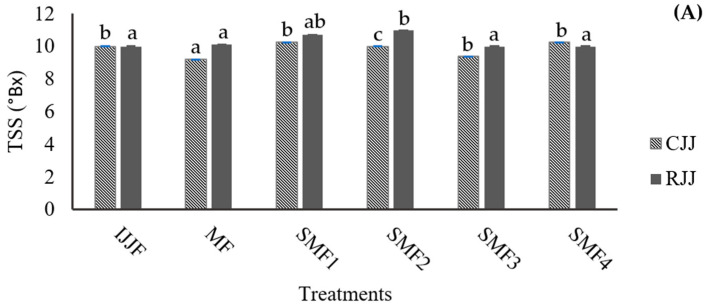

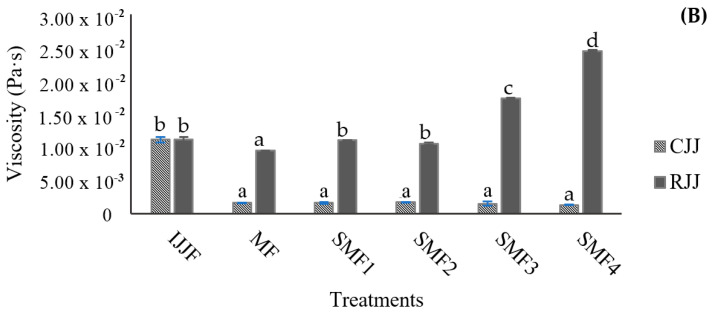
Effect of Sono-Microfiltration (SMF) on (**A**) total soluble solids (TSS) and (**B**) viscosity in jackfruit juice. IJJA: fed initial jackfruit juice, CJJ: clarified jackfruit juice, RJJ: retained jackfruit juice. Different letters show statistically significant differences.

In pulpy juices with high turbidity and a significant amount of insoluble solids, the filtration process can be hindered as the membrane pores quickly become clogged by fouling, resulting in low flow rates and yields. The particles and macromolecules retained by the membrane have a direct relationship with the turbidity of the permeate juice, which contributes to its clarification. The turbidity values recorded for both the CJJ and RJJ confirm that the microfiltration membrane effectively retained the suspended solids in the juice (Figure 8). The initial juice turbidity was 11,533 NTU, with a reduction of 99.9% after the SMF process, while the retained juices exhibited a 1.6-fold increase in turbidity when applying the MF. This indicates that it was possible to concentrate the juice up to 2.1 times in these first trials of the SMF2 treatment. The TSS concentration (Figure 9A) showed equilibrium with a difference of ±1 °Brix because these compounds, generally of lower molecular weight, passed through the microfiltration membrane’s pores and facilitated an exchange of solids between the RJJ and the CJJ due to the osmotic pressure present in the IJJ. The viscosity of the RJJ (Figure 9B) increased in treatments where an LFUS application rest time of 5 min was applied (SMF3: 0.2 min ON—5 min OFF—30% amplitude and SMF4: 1 min ON—5 min OFF—40% amplitude), leading to a higher concentration of insoluble solids on the membrane. For the CJJ, a viscosity reduction of 84.9 to 88.5% was observed, with no statistically significant differences among the evaluated treatments.

The biological properties and bioavailability of phenolic compounds depend mainly on the food matrix; transformation processes can alter the concentration of these compounds. Microfiltration (MF) is a technology that has been applied to obtain clarified beverages with stable polyphenol concentrations due to their low molecular weight (<3000 Da), which allows their passage through the membrane [58]. However, it is crucial to identify the effect of cavitation applied in the SMF pilot on the stability of the bioactive compounds that need to be preserved in functional beverages, such as total soluble polyphenols (TSP), in the various experimental treatments with the application of LFUS.

**Figure 10:**
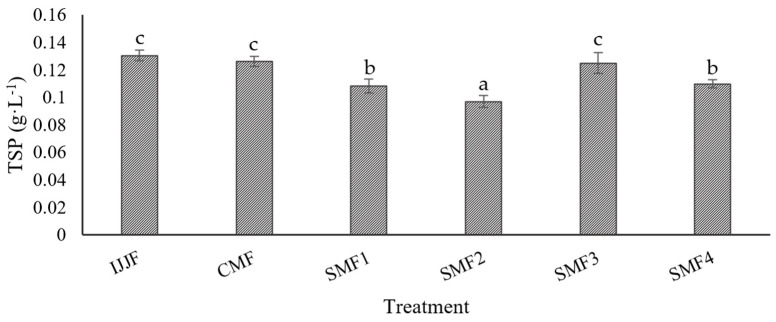
Total soluble phenols (TSP) in clarified jackfruit juice by Sono-Microfiltration (SMF) compared with the raw juice (IJJF). Different letters show statistically significant differences.

An evaluation of MF’s effect without applying LFUS on TSP concentration showed a 3% reduction (Figure 10). However, LFUS caused degradation of compounds in CJJ ranging from 0.6 and 22.2% compared to IJJF. The LFUS conditions that most affected the TSP concentration were those used in SMF2, followed by SMF1, SMF4, and SMF3, demonstrating that ultrasonic pulses with a lower frequency of application (1 min OFF) enhance the degradation of these compounds. The decrease in TSP was directly influenced by the ultrasonic energy generated in the juice during recirculation in the SMF pilot (Table 5). SMF2 presented the highest energy (8440 J·L^−1^), which was correlated with the highest decrease in TSP (22.2%) compared to IJJF, while SMF3 caused the lowest energy (870 J·L^−1^) and, therefore, the least reduction in these compounds (0.6%). The reduction of polyphenols by ultrasound has been reported due to the potential induction of free radical formation in the liquid medium, which could trigger sonochemical reactions (energy carried by sound waves to provoke and accelerate chemical reactions), thus causing the oxidation and degradation of the compounds [59]. Consequently, the decrease in antioxidant capacity generated by LFUS is attributed to the sonolysis in the liquid medium, an oxidation process that degrades organic compounds through various reactions, including the generation of highly reactive hydroxyl radicals (OH) that contribute to the degradation of compounds such as polyphenols. Acoustic cavitation, caused by sound waves, creates small bubbles in the liquid that collapse, release energy, and increase the reactivity of OH radicals [60]. It was observed that the TSP content is not significantly affected when short pulse times are applied (0.2 min ON) and when longer rest times are used (5 min OFF). Therefore, it is proposed that the optimal LFUS operating conditions to maintain a stable TSP concentration is 0.2 min ON—5 min OFF with a 30% amplitude.

Madrona et al. [61] evaluated the potential of a membrane process integrated with an ultrasonic bath to purify polyphenols and genipin components from genipap fruit extract. The process used a 0.22 µm ceramic membrane, a transmembrane pressure of 0.5 bar at 25 °C, and a 40 kHz US frequency, and 80% of the initial concentration was obtained in the permeate. Traditional methods such as heat treatments (e.g., pasteurization) are often necessary to ensure the microbiological stability and enzymatic deactivation of products, but they can lead to loss of vitamins, particularly vitamin C, and the degradation of compounds with antioxidant capacity, such as polyphenols [62]. Thus, the use of LFUS in an MF system helps reduce the deposition layer and process times; however, it affects the concentration of bioactive compounds such as FST. Therefore, further research is needed to optimize conditions to minimize this effect.

This is the first study in which low-frequency ultrasonic pulses are applied directly to the crankcase that supports a multichannel ceramic membrane in a crossflow microfiltration pilot. This coupling, known as Sono-Microfiltration, effectively reduces fouling in the membranes during the processing of pulpy jackfruit juice, resulting in shorter processing times compared to traditional tangential filtration. Additionally, the study identified the maximum ultrasonic intensity required to preserve phenolic compounds in the clarified juice. The results demonstrate that Sono-Microfiltration is a promising technique for improving the performance of industrial processes.

## 4. Conclusions

For the first time, crossflow microfiltration combined with the application of low-frequency ultrasound directly to the feed stream in the filtration membrane, referred to as Sono-Microfiltration, has shown significant potential for processing highly pulpy fruit juices such as jackfruit.

It was identified that the 2.54 cm ultrasonic probe, coupled with the Sono-Microfiltration system, radiated ultrasonic energy to a maximum distance of 20 cm. A maximum amplitude of 40% (ultrasonic power and intensity equal to or less than 26.5–28.5 W·cm^2^ and power of 130–140 W) was established to avoid damage to the TiO_2_ multichannel tubular ceramic membrane and preserve its useful life.

The evaluation of the operating conditions on filtration performance showed that a viscosity of 0.011 ± 0.002 Pa·s, ultrasonic pulses of 1 min ON with an application frequency of 5 min OFF, and an amplitude of 30% are optimal for achieving higher permeate flux. Additionally, using 0.2 min ON—5 min OFF and a 30% amplitude helps to preserve total soluble phenols in the clarified juice.

It was demonstrated that by varying process conditions, Sono-Microfiltration allows an increase in the process’s efficiency, which is reflected in a reduction in operation time by up to 50%, to achieve the same volumetric reduction ratio and preserve compounds of interest in the permeate. Therefore, Sono-Microfiltration could be viewed as an alternative process that can be applied to other barofiltration processes. This new configuration of the low-frequency ultrasound membrane module allows the development of new studies with applications in various areas at pilot and industrial scales.

This is the first study to demonstrate the potential of coupling an ultrasonic probe with a direct effect on the feed flow to the microfiltration membrane at the pilot level. The results are promising; however, further studies are required to validate this potential in various raw materials and industrial areas.

## 5. Patents

Ortiz-Basurto R.I.; Miramontes Escobar H.E.; Casillas González, R; Chacón López M.A.; Montalvo González E. (2024). Mexican Institute of Industrial Property (IMPI). Patent registration application: MX/a/2024/007272. Folio: MX/E/2024/041683.

## Figures and Tables

**Table 1:** Experimental conditions for the application of ultrasonic pulses in preventive mode.

Treatment	Operating Conditions in SMF Pilot
SMF1	0.2 min ON—1 min OFF—40% amplitude LFUS
SMF2	1 min ON—1 min OFF—30% amplitude LFUS
SMF3	0.2 min ON—5 min OFF—30% amplitude LFUS
SMF4	1 min ON—5 min OFF—40% amplitude LFUS

SMF: Sono-Microfiltration, LFUS: low-frequency ultrasound, ON: LFUS activity, OFF: without LFUS activity.

**Table 2:** Effect of amplitude US and two LFUS probes on the changes in power (W) and USI at different distances of the US probe.

Amplitude LFUS (%)	Power (W)		Energy Irradiation Distance (cm)		USI (W·cm^2^)	
	1.27 cm	2.54 cm	1.27 cm	2.54 cm	1.27 cm	2.54 cm
20	57.0 ± 0.7 ^aB^	42.2 ± 1.0 ^aA^	9.5 ± 0.7 ^aA^	8.0 ± 0.1 ^aA^	45.2 ± 0.6 ^aB^	9.4 ± 0.2 ^aA^
40	75.5 ± 4.2 ^bB^	66.7 ± 1.0 ^bB^	18.2 ± 0.3 ^bB^	10.0 ± 0.1 ^aA^	59.9 ± 3.4 ^bB^	14.8 ± 0.1 ^bA^
60	86.5 ± 0.9 ^cA^	95.7 ± 0.3 ^cB^	20.0 ± 0.1 ^bA^	19.5 ± 0.7 ^bA^	68.6 ± 0.1 ^cB^	21.3 ± 0.1 ^cA^
80	128.7 ± 1.7 ^dA^	129.5 ± 1.4 ^dA^	20.0 ± 0.1 ^bA^	20.0 ± 0.1 ^bA^	102.2 ± 1.4 ^dB^	28.8 ± 0.3 ^dA^
100	159.5 ± 1.4 ^eA^	160.0 ± 3.5 ^eA^	20.0 ± 0.1 ^bA^	20.0 ± 0.1 ^bA^	126.6 ± 1.1 ^eB^	35.6 ± 0.8 ^eA^

Lowercase letters indicate statistical differences (*p* < 0.05) between treatments in triplicate per ultrasonic probe, and uppercase letters indicate differences between probes. USI: Ultrasonic intensity, LFUS: low-frequency ultrasound.

**Table 3:** Hermia model in the Sono-Microfiltration (SMF) process.

SMF Process	Correlation Coefficients (R^2^)			
	CB	IB	SB	CF
MF	0.5385	0.6945	0.6218	0.7775
SMF1	0.7189	0.9147	0.8424	0.9577
SMF2	0.8101	0.9798	0.9157	0.9884
SMF3	0.6934	0.9202	0.8250	0.9915
SMF4	0.7255	0.9413	0.8593	0.9797

CB: complete block, IB: intermediate block, SB: Standard block, CF: formation of the deposition layer, MF: Microfiltration.

**Table 4:** Physicochemical characterization of jackfruit pulp and Sono-Microfiltration (SMF) products.

Treatment		Moisture (%)_WB_	pH	TA (%)_WB_	TRS (g·kg^−1^)_WB_
Jackfruit pulp		77.46 ± 2.9 ^b^	5.0 ± 0.1 ^a^	0.25 ± 0.03 ^d^	15.50 ± 1.0 ^c^
Feeding initial jackfruit juice (1:2 pulp/water ratio)		88.6 ± 0.6 ^b^	4.7 ± 0.1 ^a^	0.20 ± 0.01 ^c^	12.7 ± 0.2 ^b^
Retained jackfruit juice	MF	89.3 ± 0.3 ^b^	4.7 ± 0.1 ^a^	0.20 ± 0.01 ^c^	12.6 ± 0.1 ^b^
	SMF1	89.0 ± 0.7 ^b^	4.7 ± 0.1 ^a^	0.21 ± 0.01 ^c^	12.8 ± 0.1 ^b^
	SMF 2	87.5 ± 0.2 ^b^	4.7 ± 0.1 ^a^	0.22 ± 0.01 ^c^	12.8 ± 0.1 ^b^
	SMF 3	85.4 ± 1.6 ^b^	4.7 ± 0.1 ^a^	0.20 ± 0.01 ^c^	12.4 ± 0.1 ^b^
	SMF 4	87.8 ± 0.7 ^b^	4.7 ± 0.1 ^a^	0.22 ± 0.01 ^c^	12.6 ± 0.2 ^b^
Clarified jackfruit juice	MF	91.6 ± 0.5 ^a^	4.7 ± 0.1 ^a^	0.14 ± 0.01 ^a^	11.7 ± 0.1 ^b^
	SMF 1	91.9 ± 0.6 ^a^	4.7 ± 0.1 ^a^	0.16 ± 0.03 ^b^	10.8 ± 0.1 ^a^
	SMF 2	92.7 ± 0.6 ^a^	4.7 ± 0.1 ^a^	0.17 ± 0.01 ^b^	10.9 ± 0.2 ^a^
	SMF 3	91.6 ± 0.5 ^a^	4.7 ± 0.1 ^a^	0.17 ± 0.01 ^b^	10.9 ± 0.1 ^a^
	SMF 4	90.5 ± 0.6 ^a^	4.7 ± 0.1 ^a^	0.17 ± 0.01 ^b^	11.5 ± 0.2 ^b^

The results are expressed as the mean ± standard deviation (*p* < 0.05) between each determination. TA: titratable acidity; TRS: total reducing sugars; _WB_: wet basis. Different letters between columns show statistically significant differences. MF: Cross Flow Microfiltration.

**Table 5:** Ultrasonic energy generated during Sono-Microfiltration.

Operating Conditions	USE (J·L^−1^)
SMF1	5170 ± 110
SMF2	8440 ± 70
SMF3	870 ± 90
SMF4	4580 ± 110

USE: ultrasound total energy, SMF: Sono-Microfiltration.

## Data Availability

The original contributions presented in the study are included in the article, further inquiries can be directed to the corresponding author.

## Abbreviations

MF	Microfiltration
LFUS	Low-frequency ultrasound
US	Ultrasound
USI	Ultrasound intensity
USE	Ultrasound energy
SMF	Sono-Microfiltration
JP	Jackfruit pulp
JJ	Jackfruit juice
HJP	Hydrolyzed jackfruit pulp
IJJF	Initial jackfruit juice
CJJ	Clarified jackfruit juice
RJJ	Retained jackfruit juice
VRR	Volumetric reduction ratio
TMP	Transmembrane pressure (bar)
Jp	Permeate flux (L·h^−1^·m^−2^)
U	Crossflow velocity in membrane channels (m·s^−1^)
TSS	Total soluble solids (°Brix)
TA	Titratable acidity (g·kg^−1^)
NTU	Nephelometric Turbidity Units
TSP	Total soluble phenols
GAE	Gallic acid equivalent

## References

[1] Schnabel L., Kesse-Guyot E., Allès B., Touvier M., Srour B., Hercberg S., Buscail C., Julia C. (2019). Association between Ultraprocessed Food Consumption and Risk of Mortality among Middle-aged Adults in France. JAMA Intern. Med..

[2] Szwacka-Mokrzycka J., Kociszewski M. (2019). Directions of Functional Food Market Development in Light of New Consumer Trends. Acta Sci. Pol. Oeconomia.

[3] Di Corcia S., Dhuique-Mayer C., Dornier M. (2020). Concentrates from citrus juice obtained by crossflow microfiltration: Guidance of the process considering carotenoid bioaccessibility. Innov. Food Sci. Emerg. Technol..

[4] Morelli R., Conidi C., Tundis R., Loizzo M.R., D’avella M., Timpone R., Cassano A. (2022). Production of High-Quality Red Fruit Juices by Athermal Membrane Processes. Molecules.

[5] Bhattacharjee C., Saxena V.K., Dutta S. (2017). Fruit juice processing using membrane technology: A review. Innov. Food Sci. Emerg. Technol..

[6] Pradhan M., Johir M.A.H., Kandasamy J., Ratnaweera H., Vigneswaran S. (2022). Effects of Viscosity on Submerged Membrane Microfiltration Systems. Membranes.

[7] Perreault V., Gouin N., Bérubé A., Villeneuve W., Pouliot Y., Doyen A. (2021). Effect of pectinolytic enzyme pretreatment on the clarification of cranberry juice by ultrafiltration. Membranes.

[8] Terán Hilares R., Singh I., Tejada Meza K., Colina Andrade G.J., Pacheco Tanaka D.A. (2022). Alternative methods for cleaning membranes in water and wastewater treatment. Water Environ. Res..

[9] Wang T., Wang N., Li N., Ji X., Zhang H., Yu D., Wang L. (2021). Effect of high-intensity ultrasound on the physicochemical properties, microstructure, and stability of soy protein isolate-pectin emulsion. Ultrason. Sonochem..

[10] Chavan P., Sharma P., Sharma S.R., Mittal T.C., Jaiswal A.K. (2022). Application of High-Intensity Ultrasound to Improve Food Processing Efficiency: A Review. Foods.

[11] Hasan M.R., Che Abdullah C.A., Nor Afizah M., Mohd Ghazali M.S., Noranizan M.A. (2023). Efficacy of ultrasonic cleaning on cockle shells. J. Food Eng..

[12] Tzanakis I., Lebon G.S.B., Eskin D.G., Pericleous K.A. (2017). Characterizing the cavitation development and acoustic spectrum in various liquids. Ultrason. Sonochem..

[13] Rosales Pérez A., Esquivel Escalante K. (2024). The Evolution of Sonochemistry: From the Beginnings to Novel Applications. Chempluschem.

[14] Wang S.Y., Young S. (2022). Strategies for Mitigating MBR Membrane Biofouling. J. Environ. Inform. Lett..

[15] Shaik L., Chakraborty S. (2022). Nonthermal pasteurization of pineapple juice: A review on the potential of achieving microbial safety and enzymatic stability. Compr. Rev. Food Sci. Food Saf..

[16] Navarrete-Solis A. (2019). Study of the effect of ultrasound coupled to a tangential filtration pilot on the clarification of jackfruit juice (*Artocarpus heterophyllus* L.). Ph.D. Dissertation.

[17] Bayevsky M. (2004). Systems and Methods for Ultrasonic Cleaning of Cross-Flow Membrane Filters. U.S. Patent.

[18] Hengl N., Pignon F., Gondrexon N., Baup S., Yao J.I. (2019). Liquid Filtration Device Comprising an Ultrasound Emission Module. U.S. Patent.

[19] Vadoothker A.R. (2001). Ultrasound-Assited Filtration System. U.S. Patent.

[20] Whitaker S. (1986). Flow in porous media I: A theoretical derivation of Darcy’s law. Transp. Porous Media.

[21] Dahdouh L., Wisniewski C., Kapitan-Gnimdu A., Servent A., Dornier M., Delalonde M. (2015). Identification of relevant physicochemical characteristics for predicting fruit juices filterability. Sep. Purif. Technol..

[22] Vaillant F., Pérez A.M., Acosta O., Dornier M. (2008). Turbidity of pulpy fruit juice: A key factor for predicting cross-flow microfiltration performance. J. Memb. Sci..

[23] Hermia J. (1982). Constant Pressure Blocking Filtration Laws—Application To Power-law Non-newtonian Fluids. Inst. Chem. Eng. Trans..

[24] Pereira G.L.D., Cardozo-Filho L., Jegatheesan V., Guirardello R. (2023). Generalization and Expansion of the Hermia Model for a Better Understanding of Membrane Fouling. Membranes.

[25] Juárez-Trujillo N., González-Avila C., Beristain-Guevara C.I., Mendoza-López M.R., Pascual-Pineda L.A., Jiménez-Fernández M. (2023). Nutritional, physicochemical and antioxidant properties of sprouted and fermented beverages made from Phalaris canariensis seed. Int. J. Food Sci. Technol..

[26] Aghapour Aktij S., Taghipour A., Rahimpour A., Mollahosseini A., Tiraferri A. (2020). A critical review on ultrasonic-assisted fouling control and cleaning of fouled membranes. Ultrasonics.

[27] Monteiro S.H.M.C., Silva E.K., Guimarães J.T., Freitas M.Q., Meireles M.A.A., Cruz A.G. (2020). High-intensity ultrasound energy density: How different modes of application influence the quality parameters of a dairy beverage. Ultrason. Sonochem..

[28] Shi X., Tal G., Hankins N.P., Gitis V. (2014). Fouling and cleaning of ultrafiltration membranes: A review. J. Water Process Eng..

[29] Huang B., Zhao K., Zhang Z., Liu F., Hu H., Pan S. (2018). Changes on the rheological properties of pectin-enriched mango nectar by high intensity ultrasound. LWT Food Sci. Technol..

[30] Chen L., Chen L., Zhu K., Bi X., Xing Y., Che Z. (2020). The effect of high-power ultrasound on the rheological properties of strawberry pulp. Ultrason. Sonochem..

[31] El Rayess Y., Albasi C., Bacchin P., Taillandier P., Raynal J., Mietton-Peuchot M., Devatine A. (2011). Cross-flow microfiltration applied to oenology: A review. J. Memb. Sci..

[32] Jang H., Kang S., Kim J. (2024). Identification of Membrane Fouling with Greywater Filtration by Porous Membranes: Combined Effect of Membrane Pore Size and Applied Pressure. Membranes.

[33] Urošević T., Povrenović D., Vukosavljević P., Urošević I., Stevanović S. (2017). Recent developments in microfiltration and ultrafiltration of fruit juices. Food Bioprod. Process.

[34] Hammad I., Dornier M., Lebrun M., Maraval I., Poucheret P., Dhuique-Mayer C. (2022). Impact of crossflow microfiltration on aroma and sensory profiles of a potential functional citrus-based food. J. Sci. Food Agric..

[35] Vaillant F., Millan A., Dornier M., Decloux M., Reynes M. (2001). Strategy for economical optimization of the clarification of pulpy fruit juices using crossflow microfiltration. J. Food Eng..

[36] Swami S.B., Thakor N.J., Wagh S.S. (2013). Effect of temperature on viscosity of kokum, karonda, mango pulp and cashew apple syrup. Agric. Eng. Int. CIGR J..

[37] Costa M.G.M., Fonteles T.V., de Jesus A.L.T., Almeida F.D.L., de Miranda M.R.A., Fernandes F.A.N., Rodrigues S. (2013). High-Intensity Ultrasound Processing of Pineapple Juice. Food Bioprocess. Technol..

[38] Salehi F. (2020). Physicochemical characteristics and rheological behaviour of some fruit juices and their concentrates. J. Food Meas. Charact..

[39] Lamminen M.O., Walker H.W., Weavers L.K. (2004). Mechanisms and factors influencing the ultrasonic cleaning of particle-fouled ceramic membranes. J. Memb. Sci..

[40] Yusaf T., Al-Juboori R.A. (2014). Alternative methods of microorganism disruption for agricultural applications. Appl. Energy.

[41] Chen D. (2005). Ultrasonic control of ceramic membrane fouling caused by natural organic matter and silica particles. J. Membr. Sci..

[42] Chen D., Weavers L.K., Walker H.W. (2006). Ultrasonic control of ceramic membrane fouling by particles: Effect of ultrasonic factors. Ultrason. Sonochem..

[43] Cakl J., Bauer I., Doleček P., Mikulášek P. (2000). Effects of backflushing conditions on permeate flux in membrane crossflow microfiltration of oil emulsion. Desalination.

[44] Maskooki A., Kobayashi T., Mortazavi S.A., Maskooki A. (2008). Effect of low frequencies and mixed wave of ultrasound and EDTA on flux recovery and cleaning of microfiltration membranes. Sep. Purif. Technol..

[45] Kobayashi T., Kobayashi T., Hosaka Y., Fujii N. (2003). Ultrasound-enhanced membrane-cleaning processes applied water treatments: Influence of sonic frequency on filtration treatments. Ultrasonics.

[46] Muthukumaran S., Kentish S.E., Ashokkumar M., Stevens G.W. (2005). Mechanisms for the ultrasonic enhancement of dairy whey ultrafiltration. J. Memb. Sci..

[47] Mores W.D., Davis R.H. (2002). Direct observation of membrane cleaning via rapid backpulsing. Desalination.

[48] Mohammadi T., Madaeni S.S., Moghadam M.K. (2003). Investigation of membrane fouling. Desalination.

[49] Park J.Y., Lee H.C., Cho J.H. (2008). Effect of Water-Back-Flushing Time and Period in Advanced Water Treatment System by Ceramic Microfiltration. https://db.koreascholar.com/Article/Detail/244601.

[50] Ranasinghe R.A.S.N., Maduwanthi S.D.T., Marapana R.A.U.J. (2019). Nutritional and Health Benefits of Jackfruit (*Artocarpus heterophyllus* Lam.): A Review. Int. J. Food Sci..

[51] Goswami C., Chacrabati R. (2015). Jackfruit (*Artocarpus heterophylus*). Nutritional Composition of Fruit Cultivars.

[52] Galvez L., Dizon E. (2017). Physico-chemical and funtional properties of two jackfruit (*Artocarpus heterophyllus* Lam.) varieties in Eastern Visayas, Philippines. Ann. Trop. Res..

[53] Navarrete-Solis A., Hengl N., Ragazzo-Sánchez J.A., Baup S., Calderón-Santoyo M., Pignon F., López-García U.M., Ortiz-Basurto R.I. (2020). Rheological and physicochemical stability of hydrolyzed jackfruit juice (*Artocarpus heterophyllus* L.) processed by spray drying. J. Food Sci. Technol..

[54] Souza M.A., Bonomo R.C.F., Fontan R.C.I., Minim L.A., Coimbra J.S.D.R. (2011). Thermophysical properties of jackfruit pulp affected by changes in moisture content and temperature. J. Food Process Eng..

[55] Aseef R.M., Manikandan K., Kavino M., Vijayakumar R.M., Ganesan N.M. (2017). Biochemical evaluation of local genotypes of jackfruit (*Artocarpus heterophyllus* Lam.) in Pudukkotai District. J. Pharmacogn. Phytochem..

[56] Pebbuli A., Bauri F.K. (2018). Assessment of Different Quality Characters of Twenty Jackfruit Genotypes under New Alluvial Zone of West Bengal. Int. J. Curr. Microbiol. Appl. Sci..

[57] Fernández García L., Riera Rodríguez F.A. (2014). Combination of microfiltration and heat treatment for ESL milk production: Impact on shelf life. J. Food Eng..

[58] Montenegro-Landívar M.F., Tapia-Quirós P., Vecino X., Reig M., Granados M., Farran A., Cortina J.L., Saurina J., Valderrama C. (2022). Recovery of Natural Polyphenols from Spinach and Orange By-Products by Pressure-Driven Membrane Processes. Membranes.

[59] M’hiri N., Ioannou I., Mihoubi Boudhrioua N., Ghoul M. (2015). Effect of different operating conditions on the extraction of phenolic compounds in orange peel. Food Bioprod. Process.

[60] Abreu-Naranjo R., Arteaga-Crespo Y., Bravo-Sánchez L.R., Pérez-Quintana M.L., García-Quintana Y. (2020). Optimizaciòn de extraíbles totales a partir de corteza de Maytenus macrocarpa asistida por ultrasonido mediante metodología de superficie de respuesta. Afinidad.

[61] Madrona G.S., Terra N.M., Filho U.C., Magalhães F.D.S., Cardoso V.L., Reis M.H.M. (2019). Purification of phenolic compounds from genipap (*Genipa americana* L.) extract by the ultrasound assisted ultrafiltration process. Acta Sci. Technol..

[62] Cisse M., Vaillant F., Perez A., Dornier M., Reynes M. (2005). The quality of orange juice processed by coupling crossf low microfiltration and osmotic evaporation. Int. J. Food Sci. Technol..

